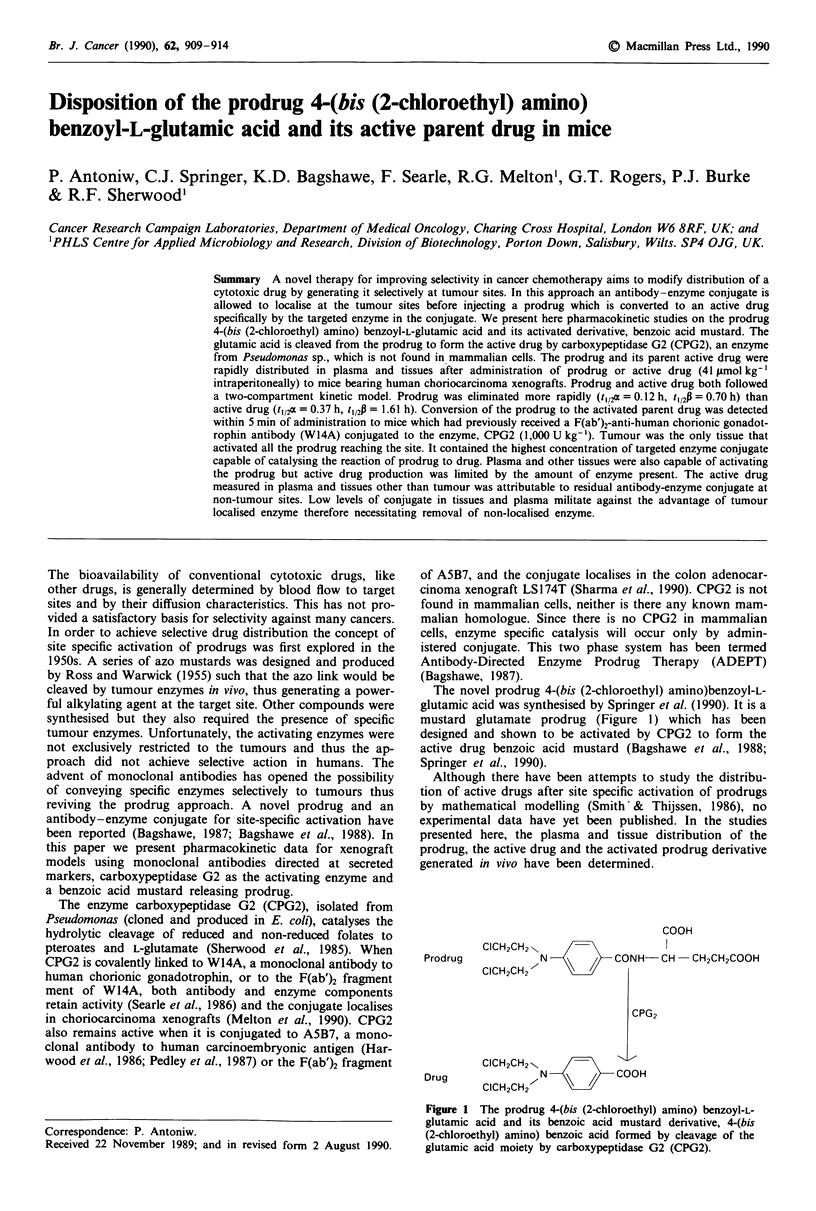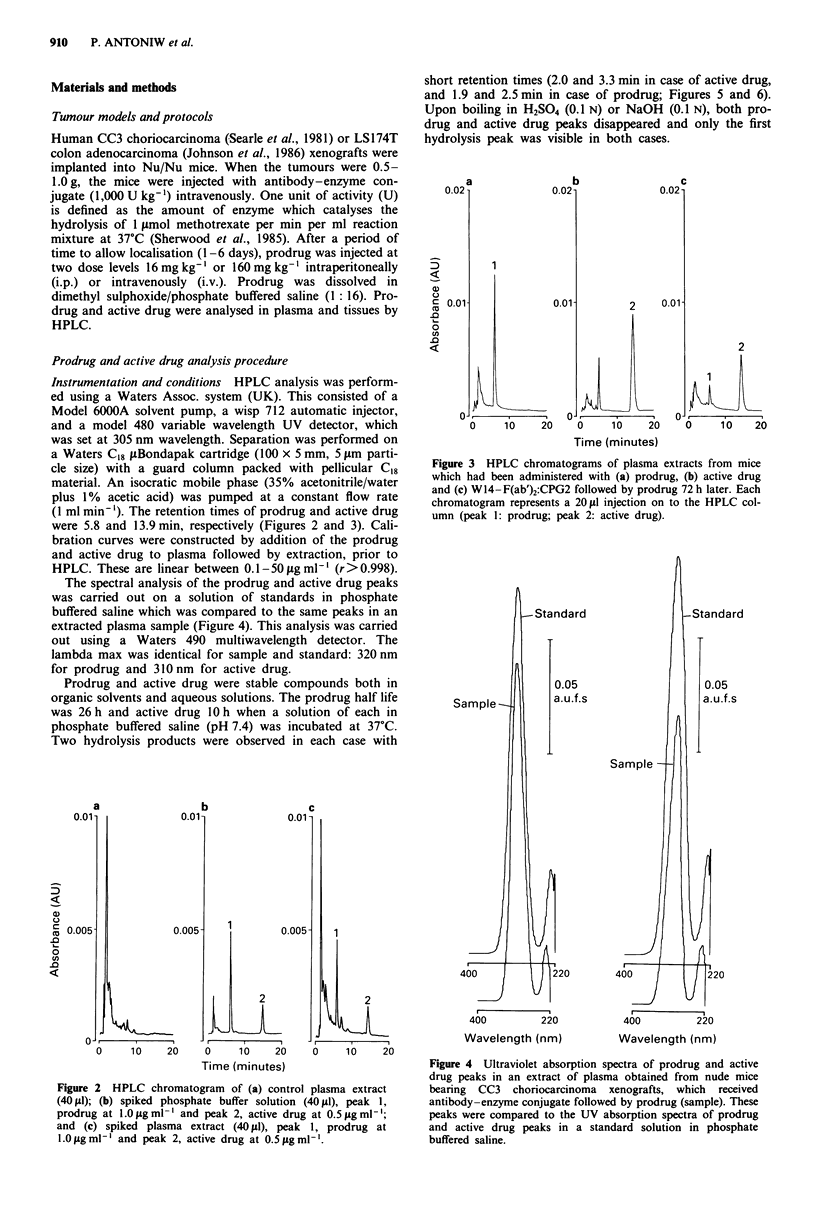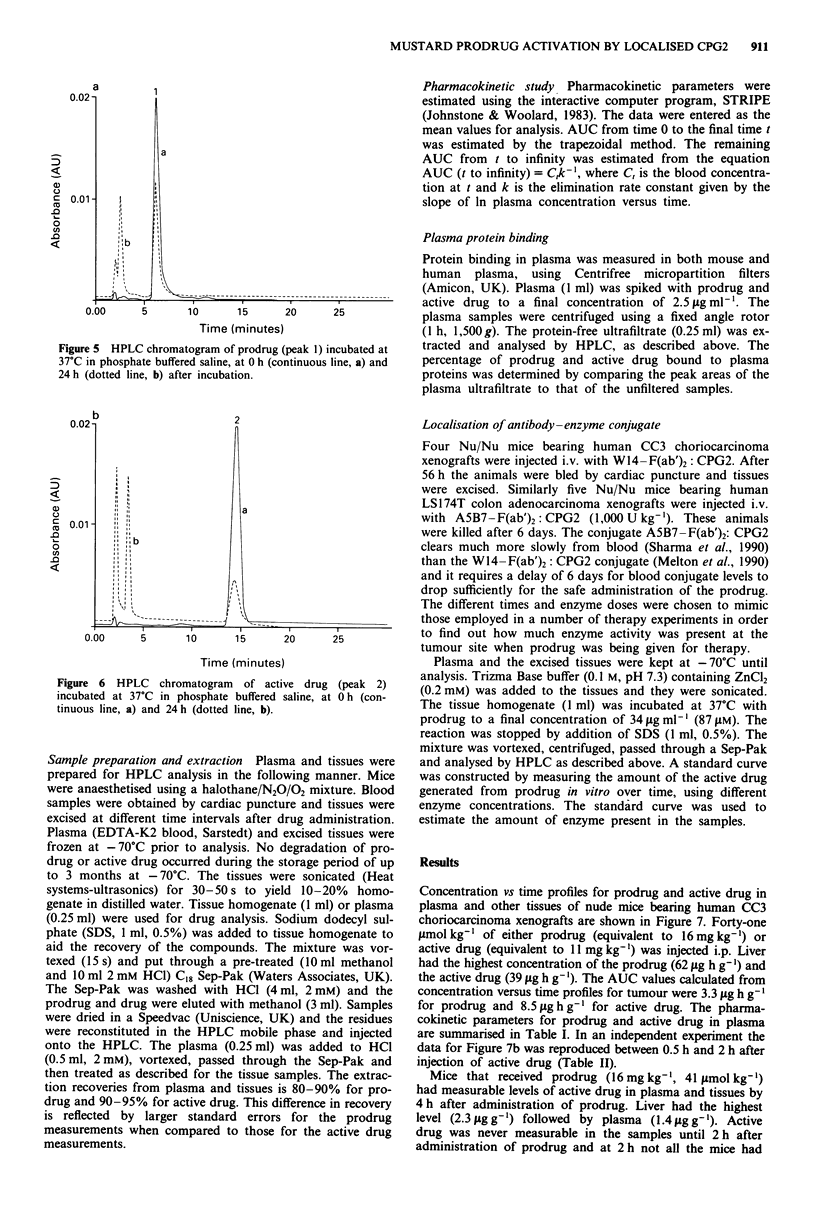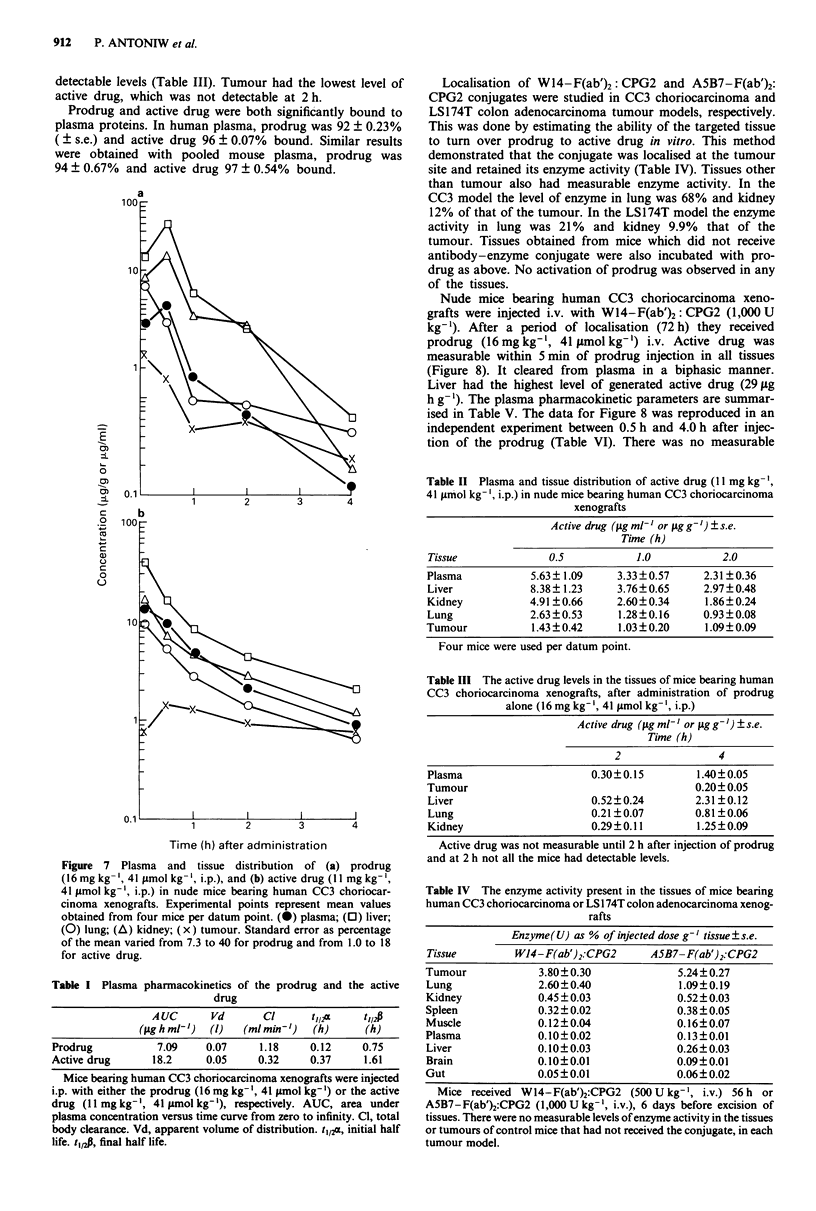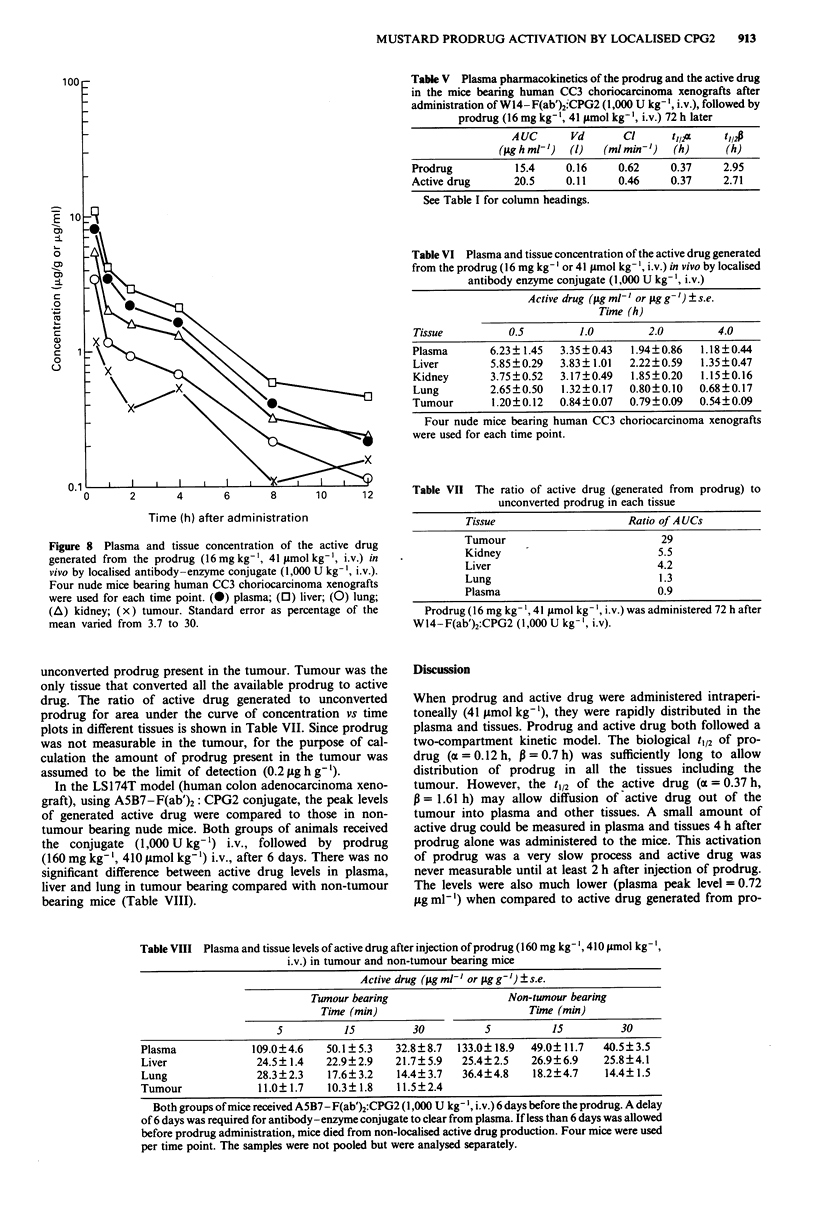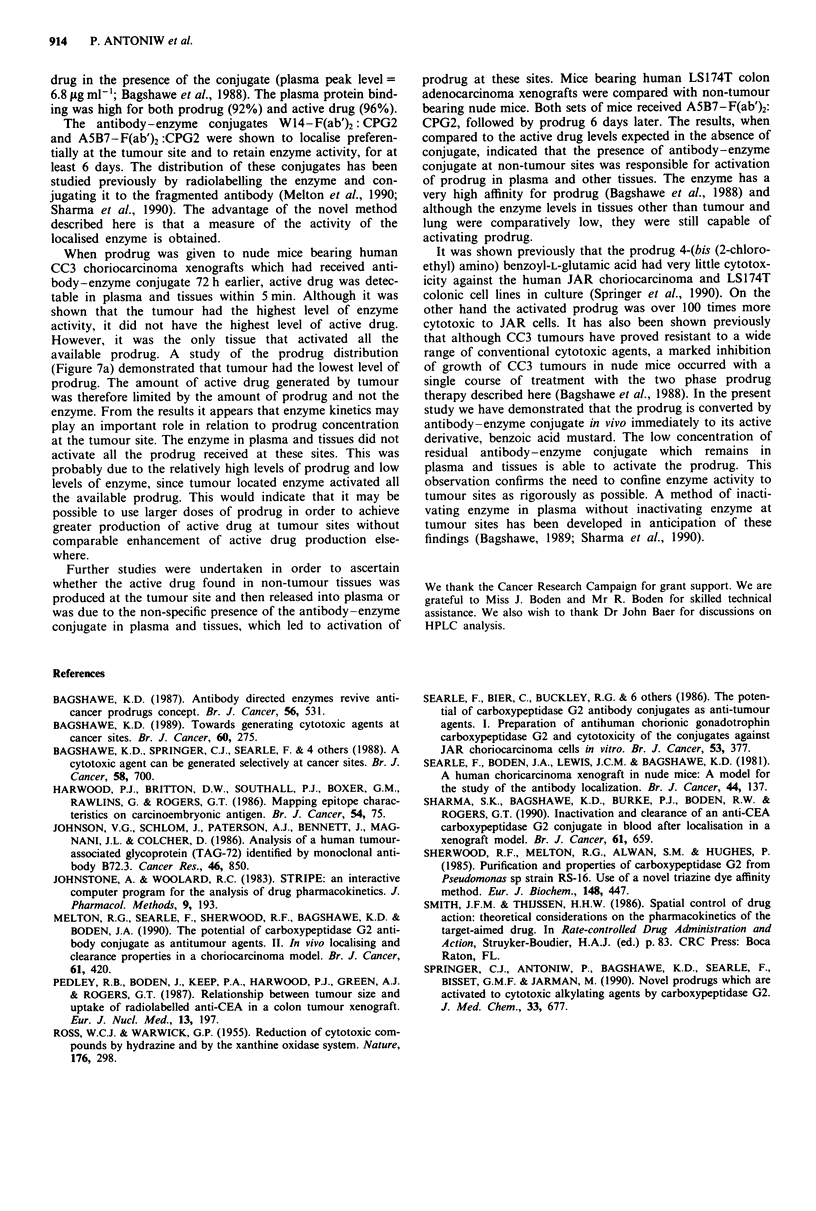# Disposition of the prodrug 4-(bis (2-chloroethyl) amino) benzoyl-L-glutamic acid and its active parent drug in mice.

**DOI:** 10.1038/bjc.1990.407

**Published:** 1990-12

**Authors:** P. Antoniw, C. J. Springer, K. D. Bagshawe, F. Searle, R. G. Melton, G. T. Rogers, P. J. Burke, R. F. Sherwood

**Affiliations:** Department of Medical Oncology, Charing Cross Hospital, London, UK.

## Abstract

A novel therapy for improving selectivity in cancer chemotherapy aims to modify distribution of a cytotoxic drug by generating it selectively at tumour sites. In this approach an antibody-enzyme conjugate is allowed to localise at the tumour sites before injecting a prodrug which is converted to an active drug specifically by the targeted enzyme in the conjugate. We present here pharmacokinetic studies on the prodrug 4-(bis (2-chloroethyl) amino) benzoyl-L-glutamic acid and its activated derivative, benzoic acid mustard. The glutamic acid is cleaved from the prodrug to form the active drug by carboxypeptidase G2 (CPG2), an enzyme from Pseudomonas sp., which is not found in mammalian cells. The prodrug and its parent active drug were rapidly distributed in plasma and tissues after administration of prodrug or active drug (41 mumol kg-1 intraperitoneally) to mice bearing human choriocarcinoma xenografts. Prodrug and active drug both followed a two-compartment kinetic model. Prodrug was eliminated more rapidly (t1/2 alpha = 0.12 h, t1/2 beta = 0.70 h) than active drug (t1/2 alpha = 0.37 h, t1/2 beta = 1.61 h). Conversion of the prodrug to the activated parent drug was detected within 5 min of administration to mice which had previously received a F(ab')2-anti-human chorionic gonadotrophin antibody (W14A) conjugated to the enzyme, CPG2 (1,000 U kg-1). Tumour was the only tissue that activated all the prodrug reaching the site. It contained the highest concentration of targeted enzyme conjugate capable of catalysing the reaction of prodrug to drug. Plasma and other tissues were also capable of activating the prodrug but active drug production was limited by the amount of enzyme present. The active drug measured in plasma and tissues other than tumour was attributable to residual antibody-enzyme conjugate at non-tumour sites. Low levels of conjugate in tissues and plasma militate against the advantage of tumour localised enzyme therefore necessitating removal of non-localised enzyme.


Br. J. Cancer (1990), 62, 909-914 © Macmillan Press Ltd., 1990

### Disposition of the prodrug 4-(*bis* (2-chloroethyl) amino) benzoyl-L-glutamic acid and its active parent drug in mice

P. Antoniw, C.J. Springer, K.D. Bagshawe, F. Searle, R.G. Melton[1], G.T. Rogers, P.J. Burke & R.F. Sherwood[1]

*Cancer Research Campaign Laboratories, Department of Medical Oncology, Charing Cross Hospital, London W6 8RF, UK; and [1]PHLS Centre for Applied Microbiology and Research, Division of Biotechnology, Porton Down, Salisbury, Wilts. SP4 OJG, UK.*

**Summary** A novel therapy for improving selectivity in cancer chemotherapy aims to modify distribution of a cytotoxic drug by generating it selectively at tumour sites. In this approach an antibody-enzyme conjugate is allowed to localise at the tumour sites before injecting a prodrug which is converted to an active drug specifically by the targeted enzyme in the conjugate. We present here pharmacokinetic studies on the prodrug 4-(*bis* (2-chloroethyl) amino) benzoyl-L-glutamic acid and its activated derivative, benzoic acid mustard. The glutamic acid is cleaved from the prodrug to form the active drug by carboxypeptidase G2 (CPG2), an enzyme from *Pseudomonas* sp., which is not found in mammalian cells. The prodrug and its parent active drug were rapidly distributed in plasma and tissues after administration of prodrug or active drug (41 $\mu$mol kg$^{-1}$ intraperitoneally) to mice bearing human choriocarcinoma xenografts. Prodrug and active drug both followed a two-compartment kinetic model. Prodrug was eliminated more rapidly ($t_{1/2}\alpha = 0.12$ h, $t_{1/2}\beta = 0.70$ h) than active drug ($t_{1/2}\alpha = 0.37$ h, $t_{1/2}\beta = 1.61$ h). Conversion of the prodrug to the activated parent drug was detected within 5 min of administration to mice which had previously received a F(ab')$_2$-anti-human chorionic gonadotrophin antibody (W14A) conjugated to the enzyme, CPG2 (1,000 U kg$^{-1}$). Tumour was the only tissue that activated all the prodrug reaching the site. It contained the highest concentration of targeted enzyme conjugate capable of catalysing the reaction of prodrug to drug. Plasma and other tissues were also capable of activating the prodrug but active drug production was limited by the amount of enzyme present. The active drug measured in plasma and tissues other than tumour was attributable to residual antibody-enzyme conjugate at non-tumour sites. Low levels of conjugate in tissues and plasma militate against the advantage of tumour localised enzyme therefore necessitating removal of non-localised enzyme.

The bioavailability of conventional cytotoxic drugs, like other drugs, is generally determined by blood flow to target sites and by their diffusion characteristics. This has not provided a satisfactory basis for selectivity against many cancers. In order to achieve selective drug distribution the concept of site specific activation of prodrugs was first explored in the 1950s. A series of azo mustards was designed and produced by Ross and Warwick (1955) such that the azo link would be cleaved by tumour enzymes *in vivo*, thus generating a powerful alkylating agent at the target site. Other compounds were synthesised but they also required the presence of specific tumour enzymes. Unfortunately, the activating enzymes were not exclusively restricted to the tumours and thus the approach did not achieve selective action in humans. The advent of monoclonal antibodies has opened the possibility of conveying specific enzymes selectively to tumours thus reviving the prodrug approach. A novel prodrug and an antibody-enzyme conjugate for site-specific activation have been reported (Bagshawe, 1987; Bagshawe *et al*., 1988). In this paper we present pharmacokinetic data for xenograft models using monoclonal antibodies directed at secreted markers, carboxypeptidase G2 as the activating enzyme and a benzoic acid mustard releasing prodrug.

The enzyme carboxypeptidase G2 (CPG2), isolated from *Pseudomonas* (cloned and produced in *E. coli*), catalyses the hydrolytic cleavage of reduced and non-reduced folates to pteroates and L-glutamate (Sherwood *et al*., 1985). When CPG2 is covalently linked to W14A, a monoclonal antibody to human chorionic gonadotrophin, or to the F(ab')$_2$ fragment ment of W14A, both antibody and enzyme components retain activity (Searle *et al*., 1986) and the conjugate localises in choriocarcinoma xenografts (Melton *et al*., 1990). CPG2 also remains active when it is conjugated to A5B7, a monoclonal antibody to human carcinoembryonic antigen (Harwood *et al*., 1986; Pedley *et al*., 1987) or the F(ab')$_2$ fragment of A5B7, and the conjugate localises in the colon adenocarcinoma xenograft LS174T (Sharma *et al*., 1990). CPG2 is not found in mammalian cells, neither is there any known mammalian homologue. Since there is no CPG2 in mammalian cells, enzyme specific catalysis will occur only by administered conjugate. This two phase system has been termed Antibody-Directed Enzyme Prodrug Therapy (ADEPT) (Bagshawe, 1987).

The novel prodrug 4-(*bis* (2-chloroethyl) amino)benzoyl-L-glutamic acid was synthesised by Springer *et al*. (1990). It is a mustard glutamate prodrug (Figure 1) which has been designed and shown to be activated by CPG2 to form the active drug benzoic acid mustard (Bagshawe *et al*., 1988; Springer *et al*., 1990).

Although there have been attempts to study the distribution of active drugs after site specific activation of prodrugs by mathematical modelling (Smith & Thijssen, 1986), no experimental data have yet been published. In the studies presented here, the plasma and tissue distribution of the prodrug, the active drug and the activated prodrug derivative generated *in vivo* have been determined.

**Figure 1** The prodrug 4-(*bis* (2-chloroethyl) amino) benzoyl-L-glutamic acid and its benzoic acid mustard derivative, 4-(*bis* (2-chloroethyl) amino) benzoic acid formed by cleavage of the glutamic acid moiety by carboxypeptidase G2 (CPG2).

Correspondence: P. Antoniw.
Received 22 November 1989; and in revised form 2 August 1990.

### Materials and methods

### *Tumour models and protocols*

Human CC3 choriocarcinoma (Searle *et al.*, 1981) or LS174T colon adenocarcinoma (Johnson *et al.*, 1986) xenografts were implanted into Nu/Nu mice. When the tumours were 0.5–1.0 g, the mice were injected with antibody–enzyme conjugate (1,000 U $kg^{-1}$) intravenously. One unit of activity (U) is defined as the amount of enzyme which catalyses the hydrolysis of 1 μmol methotrexate per min per ml reaction mixture at 37°C (Sherwood *et al.*, 1985). After a period of time to allow localisation (1–6 days), prodrug was injected at two dose levels 16 mg $kg^{-1}$ or 160 mg $kg^{-1}$ intraperitoneally (i.p.) or intravenously (i.v.). Prodrug was dissolved in dimethyl sulphoxide/phosphate buffered saline (1 : 16). Prodrug and active drug were analysed in plasma and tissues by HPLC.

### *Prodrug and active drug analysis procedure*

*Instrumentation and conditions* HPLC analysis was performed using a Waters Assoc. system (UK). This consisted of a Model 6000A solvent pump, a wisp 712 automatic injector, and a model 480 variable wavelength UV detector, which was set at 305 nm wavelength. Separation was performed on a Waters $C_{18}$ μBondapak cartridge (100 × 5 mm, 5 μm particle size) with a guard column packed with pellicular $C_{18}$ material. An isocratic mobile phase (35% acetonitrile/water plus 1% acetic acid) was pumped at a constant flow rate (1 ml $min^{-1}$). The retention times of prodrug and active drug were 5.8 and 13.9 min, respectively (Figures 2 and 3). Calibration curves were constructed by addition of the prodrug and active drug to plasma followed by extraction, prior to HPLC. These are linear between 0.1–50 μg $ml^{-1}$ ($r > 0.998$).

The spectral analysis of the prodrug and active drug peaks was carried out on a solution of standards in phosphate buffered saline which was compared to the same peaks in an extracted plasma sample (Figure 4). This analysis was carried out using a Waters 490 multiwavelength detector. The lambda max was identical for sample and standard: 320 nm for prodrug and 310 nm for active drug.

Prodrug and active drug were stable compounds both in organic solvents and aqueous solutions. The prodrug half life was 26 h and active drug 10 h when a solution of each in phosphate buffered saline (pH 7.4) was incubated at 37°C. Two hydrolysis products were observed in each case with short retention times (2.0 and 3.3 min in case of active drug, and 1.9 and 2.5 min in case of prodrug; Figures 5 and 6). Upon boiling in $H_2SO_4$ (0.1 N) or NaOH (0.1 N), both prodrug and active drug peaks disappeared and only the first hydrolysis peak was visible in both cases.

**Figure 2** HPLC chromatogram of (**a**) control plasma extract (40 μl); (**b**) spiked phosphate buffer solution (40 μl), peak 1, prodrug at 1.0 μg $ml^{-1}$ and peak 2, active drug at 0.5 μg $ml^{-1}$; and (**c**) spiked plasma extract (40 μl), peak 1, prodrug at 1.0 μg $ml^{-1}$ and peak 2, active drug at 0.5 μg $ml^{-1}$.

**Figure 3** HPLC chromatograms of plasma extracts from mice which had been administered with (**a**) prodrug, (**b**) active drug and (**c**) W14–F(ab′)$_2$:CPG2 followed by prodrug 72 h later. Each chromatogram represents a 20 μl injection on to the HPLC column (peak 1: prodrug; peak 2: active drug).

**Figure 4** Ultraviolet absorption spectra of prodrug and active drug peaks in an extract of plasma obtained from nude mice bearing CC3 choriocarcinoma xenografts, which received antibody–enzyme conjugate followed by prodrug (sample). These peaks were compared to the UV absorption spectra of prodrug and active drug peaks in a standard solution in phosphate buffered saline.

**Figure 5** HPLC chromatogram of prodrug (peak 1) incubated at 37°C in phosphate buffered saline, at 0 h (continuous line, **a**) and 24 h (dotted line, **b**) after incubation.

**Figure 6** HPLC chromatogram of active drug (peak 2) incubated at 37°C in phosphate buffered saline, at 0 h (continuous line, **a**) and 24 h (dotted line, **b**).

*Sample preparation and extraction* Plasma and tissues were prepared for HPLC analysis in the following manner. Mice were anaesthetised using a halothane/$N_2O/O_2$ mixture. Blood samples were obtained by cardiac puncture and tissues were excised at different time intervals after drug administration. Plasma (EDTA-K2 blood, Sarstedt) and excised tissues were frozen at − 70°C prior to analysis. No degradation of prodrug or active drug occurred during the storage period of up to 3 months at − 70°C. The tissues were sonicated (Heat systems-ultrasonics) for 30–50 s to yield 10–20% homogenate in distilled water. Tissue homogenate (1 ml) or plasma (0.25 ml) were used for drug analysis. Sodium dodecyl sulphate (SDS, 1 ml, 0.5%) was added to tissue homogenate to aid the recovery of the compounds. The mixture was vortexed (15 s) and put through a pre-treated (10 ml methanol and 10 ml 2 mM HCl) $C_{18}$ Sep-Pak (Waters Associates, UK). The Sep-Pak was washed with HCl (4 ml, 2 mM) and the prodrug and drug were eluted with methanol (3 ml). Samples were dried in a Speedvac (Uniscience, UK) and the residues were reconstituted in the HPLC mobile phase and injected onto the HPLC. The plasma (0.25 ml) was added to HCl (0.5 ml, 2 mM), vortexed, passed through the Sep-Pak and then treated as described for the tissue samples. The extraction recoveries from plasma and tissues is 80–90% for prodrug and 90–95% for active drug. This difference in recovery is reflected by larger standard errors for the prodrug measurements when compared to those for the active drug measurements.

*Pharmacokinetic study* Pharmacokinetic parameters were estimated using the interactive computer program, STRIPE (Johnstone & Woolard, 1983). The data were entered as the mean values for analysis. AUC from time 0 to the final time *t* was estimated by the trapezoidal method. The remaining AUC from *t* to infinity was estimated from the equation AUC (*t* to infinity) = $C_t k^{-1}$, where $C_t$ is the blood concentration at *t* and *k* is the elimination rate constant given by the slope of ln plasma concentration versus time.

*Plasma protein binding*

Protein binding in plasma was measured in both mouse and human plasma, using Centrifree micropartition filters (Amicon, UK). Plasma (1 ml) was spiked with prodrug and active drug to a final concentration of 2.5 $\mu g\ ml^{-1}$. The plasma samples were centrifuged using a fixed angle rotor (1 h, 1,500 *g*). The protein-free ultrafiltrate (0.25 ml) was extracted and analysed by HPLC, as described above. The percentage of prodrug and active drug bound to plasma proteins was determined by comparing the peak areas of the plasma ultrafiltrate to that of the unfiltered samples.

*Localisation of antibody–enzyme conjugate*

Four Nu/Nu mice bearing human CC3 choriocarcinoma xenografts were injected i.v. with W14–$F(ab')_2$ : CPG2. After 56 h the animals were bled by cardiac puncture and tissues were excised. Similarly five Nu/Nu mice bearing human LS174T colon adenocarcinoma xenografts were injected i.v. with A5B7–$F(ab')_2$ : CPG2 (1,000 U $kg^{-1}$). These animals were killed after 6 days. The conjugate A5B7–$F(ab')_2$: CPG2 clears much more slowly from blood (Sharma *et al.*, 1990) than the W14–$F(ab')_2$ : CPG2 conjugate (Melton *et al.*, 1990) and it requires a delay of 6 days for blood conjugate levels to drop sufficiently for the safe administration of the prodrug. The different times and enzyme doses were chosen to mimic those employed in a number of therapy experiments in order to find out how much enzyme activity was present at the tumour site when prodrug was being given for therapy.

Plasma and the excised tissues were kept at − 70°C until analysis. Trizma Base buffer (0.1 M, pH 7.3) containing $ZnCl_2$ (0.2 mM) was added to the tissues and they were sonicated. The tissue homogenate (1 ml) was incubated at 37°C with prodrug to a final concentration of 34 $\mu g\ ml^{-1}$ (87 $\mu$M). The reaction was stopped by addition of SDS (1 ml, 0.5%). The mixture was vortexed, centrifuged, passed through a Sep-Pak and analysed by HPLC as described above. A standard curve was constructed by measuring the amount of the active drug generated from prodrug *in vitro* over time, using different enzyme concentrations. The standard curve was used to estimate the amount of enzyme present in the samples.

### Results

Concentration *vs* time profiles for prodrug and active drug in plasma and other tissues of nude mice bearing human CC3 choriocarcinoma xenografts are shown in Figure 7. Forty-one $\mu mol\ kg^{-1}$ of either prodrug (equivalent to 16 mg $kg^{-1}$) or active drug (equivalent to 11 mg $kg^{-1}$) was injected i.p. Liver had the highest concentration of the prodrug (62 $\mu g\ h\ g^{-1}$) and the active drug (39 $\mu g\ h\ g^{-1}$). The AUC values calculated from concentration versus time profiles for tumour were 3.3 $\mu g\ h\ g^{-1}$ for prodrug and 8.5 $\mu g\ h\ g^{-1}$ for active drug. The pharmacokinetic parameters for prodrug and active drug in plasma are summarised in Table I. In an independent experiment the data for Figure 7b was reproduced between 0.5 h and 2 h after injection of active drug (Table II).

Mice that received prodrug (16 mg $kg^{-1}$, 41 $\mu mol\ kg^{-1}$) had measurable levels of active drug in plasma and tissues by 4 h after administration of prodrug. Liver had the highest level (2.3 $\mu g\ g^{-1}$) followed by plasma (1.4 $\mu g\ g^{-1}$). Active drug was never measurable in the samples until 2 h after administration of prodrug and at 2 h not all the mice had detectable levels (Table III). Tumour had the lowest level of active drug, which was not detectable at 2 h.

Prodrug and active drug were both significantly bound to plasma proteins. In human plasma, prodrug was 92 ± 0.23% (± s.e.) and active drug 96 ± 0.07% bound. Similar results were obtained with pooled mouse plasma, prodrug was 94 ± 0.67% and active drug 97 ± 0.54% bound.

**Figure 7** Plasma and tissue distribution of (**a**) prodrug (16 mg kg$^{-1}$, 41 µmol kg$^{-1}$, i.p.), and (**b**) active drug (11 mg kg$^{-1}$, 41 µmol kg$^{-1}$, i.p.) in nude mice bearing human CC3 choriocarcinoma xenografts. Experimental points represent mean values obtained from four mice per datum point. (●) plasma; (□) liver; (○) lung; (△) kidney; (×) tumour. Standard error as percentage of the mean varied from 7.3 to 40 for prodrug and from 1.0 to 18 for active drug.

**Table I** Plasma pharmacokinetics of the prodrug and the active drug

| | *AUC* (*µg h ml*$^{-1}$) | *Vd* (*l*) | *Cl* (*ml min*$^{-1}$) | $t_{1/2}\alpha$ (*h*) | $t_{1/2}\beta$ (*h*) |
|---|---|---|---|---|---|
| Prodrug | 7.09 | 0.07 | 1.18 | 0.12 | 0.75 |
| Active drug | 18.2 | 0.05 | 0.32 | 0.37 | 1.61 |

Mice bearing human CC3 choriocarcinoma xenografts were injected i.p. with either the prodrug (16 mg kg$^{-1}$, 41 µmol kg$^{-1}$) or the active drug (11 mg kg$^{-1}$, 41 µmol kg$^{-1}$), respectively. AUC, area under plasma concentration versus time curve from zero to infinity. Cl, total body clearance. Vd, apparent volume of distribution. $t_{1/2}\alpha$, initial half life. $t_{1/2}\beta$, final half life.

Localisation of W14–F(ab')$_2$ : CPG2 and A5B7–F(ab')$_2$: CPG2 conjugates were studied in CC3 choriocarcinoma and LS174T colon adenocarcinoma tumour models, respectively. This was done by estimating the ability of the targeted tissue to turn over prodrug to active drug *in vitro*. This method demonstrated that the conjugate was localised at the tumour site and retained its enzyme activity (Table IV). Tissues other than tumour also had measurable enzyme activity. In the CC3 model the level of enzyme in lung was 68% and kidney 12% of that of the tumour. In the LS174T model the enzyme activity in lung was 21% and kidney 9.9% that of the tumour. Tissues obtained from mice which did not receive antibody–enzyme conjugate were also incubated with prodrug as above. No activation of prodrug was observed in any of the tissues.

Nude mice bearing human CC3 choriocarcinoma xenografts were injected i.v. with W14–F(ab')$_2$ : CPG2 (1,000 U kg$^{-1}$). After a period of localisation (72 h) they received prodrug (16 mg kg$^{-1}$, 41 µmol kg$^{-1}$) i.v. Active drug was measurable within 5 min of prodrug injection in all tissues (Figure 8). It cleared from plasma in a biphasic manner. Liver had the highest level of generated active drug (29 µg h g$^{-1}$). The plasma pharmacokinetic parameters are summarised in Table V. The data for Figure 8 was reproduced in an independent experiment between 0.5 h and 4.0 h after injection of the prodrug (Table VI). There was no measurable

**Table II** Plasma and tissue distribution of active drug (11 mg kg$^{-1}$, 41 µmol kg$^{-1}$, i.p.) in nude mice bearing human CC3 choriocarcinoma xenografts

| | *Active drug (µg ml$^{-1}$ or µg g$^{-1}$) ± s.e. Time (h)* | | |
|---|---|---|---|
| *Tissue* | *0.5* | *1.0* | *2.0* |
| Plasma | 5.63 ± 1.09 | 3.33 ± 0.57 | 2.31 ± 0.36 |
| Liver | 8.38 ± 1.23 | 3.76 ± 0.65 | 2.97 ± 0.48 |
| Kidney | 4.91 ± 0.66 | 2.60 ± 0.34 | 1.86 ± 0.24 |
| Lung | 2.63 ± 0.53 | 1.28 ± 0.16 | 0.93 ± 0.08 |
| Tumour | 1.43 ± 0.42 | 1.03 ± 0.20 | 1.09 ± 0.09 |

Four mice were used per datum point.

**Table III** The active drug levels in the tissues of mice bearing human CC3 choriocarcinoma xenografts, after administration of prodrug alone (16 mg kg$^{-1}$, 41 µmol kg$^{-1}$, i.p.)

| | *Active drug (µg ml$^{-1}$ or µg g$^{-1}$) ± s.e. Time (h)* | |
|---|---|---|
| | *2* | *4* |
| Plasma | 0.30 ± 0.15 | 1.40 ± 0.05 |
| Tumour | | 0.20 ± 0.05 |
| Liver | 0.52 ± 0.24 | 2.31 ± 0.12 |
| Lung | 0.21 ± 0.07 | 0.81 ± 0.06 |
| Kidney | 0.29 ± 0.11 | 1.25 ± 0.09 |

Active drug was not measurable until 2 h after injection of prodrug and at 2 h not all the mice had detectable levels.

**Table IV** The enzyme activity present in the tissues of mice bearing human CC3 choriocarcinoma or LS174T colon adenocarcinoma xenografts

| | *Enzyme(U) as % of injected dose g$^{-1}$ tissue ± s.e.* | |
|---|---|---|
| *Tissue* | *W14–F(ab')$_2$:CPG2* | *A5B7–F(ab')$_2$:CPG2* |
| Tumour | 3.80 ± 0.30 | 5.24 ± 0.27 |
| Lung | 2.60 ± 0.40 | 1.09 ± 0.19 |
| Kidney | 0.45 ± 0.03 | 0.52 ± 0.03 |
| Spleen | 0.32 ± 0.02 | 0.38 ± 0.05 |
| Muscle | 0.12 ± 0.04 | 0.16 ± 0.07 |
| Plasma | 0.10 ± 0.02 | 0.13 ± 0.01 |
| Liver | 0.10 ± 0.03 | 0.26 ± 0.03 |
| Brain | 0.10 ± 0.01 | 0.09 ± 0.01 |
| Gut | 0.05 ± 0.01 | 0.06 ± 0.02 |

Mice received W14–F(ab')$_2$:CPG2 (500 U kg$^{-1}$, i.v.) 56 h or A5B7–F(ab')$_2$:CPG2 (1,000 U kg$^{-1}$, i.v.), 6 days before excision of tissues. There were no measurable levels of enzyme activity in the tissues or tumours of control mice that had not received the conjugate, in each tumour model.

**Figure 8** Plasma and tissue concentration of the active drug generated from the prodrug (16 mg kg$^{-1}$, 41 µmol kg$^{-1}$, i.v.) *in vivo* by localised antibody–enzyme conjugate (1,000 U kg$^{-1}$, i.v.). Four nude mice bearing human CC3 choriocarcinoma xenografts were used for each time point. (●) plasma; (□) liver; (O) lung; (Δ) kidney; (×) tumour. Standard error as percentage of the mean varied from 3.7 to 30.

**Table V** Plasma pharmacokinetics of the prodrug and the active drug in the mice bearing human CC3 choriocarcinoma xenografts after administration of W14–F(ab')$_2$:CPG2 (1,000 U kg$^{-1}$, i.v.), followed by prodrug (16 mg kg$^{-1}$, 41 µmol kg$^{-1}$, i.v.) 72 h later

| | *AUC* (*µg h ml$^{-1}$*) | *Vd* (*l*) | *Cl* (*ml min$^{-1}$*) | $t_{1/2}\alpha$ (*h*) | $t_{1/2}\beta$ (*h*) |
|---|---|---|---|---|---|
| Prodrug | 15.4 | 0.16 | 0.62 | 0.37 | 2.95 |
| Active drug | 20.5 | 0.11 | 0.46 | 0.37 | 2.71 |

See Table I for column headings.

**Table VI** Plasma and tissue concentration of the active drug generated from the prodrug (16 mg kg$^{-1}$ or 41 µmol kg$^{-1}$, i.v.) *in vivo* by localised antibody enzyme conjugate (1,000 U kg$^{-1}$, i.v.)

| | *Active drug (µg ml$^{-1}$ or µg g$^{-1}$) ± s.e.* Time (h) | | | |
|---|---|---|---|---|
| *Tissue* | *0.5* | *1.0* | *2.0* | *4.0* |
| Plasma | 6.23 ± 1.45 | 3.35 ± 0.43 | 1.94 ± 0.86 | 1.18 ± 0.44 |
| Liver | 5.85 ± 0.29 | 3.83 ± 1.01 | 2.22 ± 0.59 | 1.35 ± 0.47 |
| Kidney | 3.75 ± 0.52 | 3.17 ± 0.49 | 1.85 ± 0.20 | 1.15 ± 0.16 |
| Lung | 2.65 ± 0.50 | 1.32 ± 0.17 | 0.80 ± 0.10 | 0.68 ± 0.17 |
| Tumour | 1.20 ± 0.12 | 0.84 ± 0.07 | 0.79 ± 0.09 | 0.54 ± 0.09 |

Four nude mice bearing human CC3 choriocarcinoma xenografts were used for each time point.

**Table VII** The ratio of active drug (generated from prodrug) to unconverted prodrug in each tissue

| *Tissue* | *Ratio of AUCs* |
|---|---|
| Tumour | 29 |
| Kidney | 5.5 |
| Liver | 4.2 |
| Lung | 1.3 |
| Plasma | 0.9 |

Prodrug (16 mg kg$^{-1}$, 41 µmol kg$^{-1}$, i.v.) was administered 72 h after W14–F(ab')$_2$:CPG2 (1,000 U kg$^{-1}$, i.v).

unconverted prodrug present in the tumour. Tumour was the only tissue that converted all the available prodrug to active drug. The ratio of active drug generated to unconverted prodrug for area under the curve of concentration *vs* time plots in different tissues is shown in Table VII. Since prodrug was not measurable in the tumour, for the purpose of calculation the amount of prodrug present in the tumour was assumed to be the limit of detection (0.2 µg h g$^{-1}$).

In the LS174T model (human colon adenocarcinoma xenograft), using A5B7–F(ab')$_2$ : CPG2 conjugate, the peak levels of generated active drug were compared to those in non-tumour bearing nude mice. Both groups of animals received the conjugate (1,000 U kg$^{-1}$) i.v., followed by prodrug (160 mg kg$^{-1}$, 410 µmol kg$^{-1}$) i.v., after 6 days. There was no significant difference between active drug levels in plasma, liver and lung in tumour bearing compared with non-tumour bearing mice (Table VIII).

### Discussion

When prodrug and active drug were administered intraperitoneally (41 µmol kg$^{-1}$), they were rapidly distributed in the plasma and tissues. Prodrug and active drug both followed a two-compartment kinetic model. The biological $t_{1/2}$ of prodrug ($\alpha = 0.12$ h, $\beta = 0.7$ h) was sufficiently long to allow distribution of prodrug in all the tissues including the tumour. However, the $t_{1/2}$ of the active drug ($\alpha = 0.37$ h, $\beta = 1.61$ h) may allow diffusion of active drug out of the tumour into plasma and other tissues. A small amount of active drug could be measured in plasma and tissues 4 h after prodrug alone was administered to the mice. This activation of prodrug was a very slow process and active drug was never measurable until at least 2 h after injection of prodrug. The levels were also much lower (plasma peak level = 0.72 µg ml$^{-1}$) when compared to active drug generated from pro-

**Table VIII** Plasma and tissue levels of active drug after injection of prodrug (160 mg kg$^{-1}$, 410 µmol kg$^{-1}$, i.v.) in tumour and non-tumour bearing mice

| | *Active drug (µg ml$^{-1}$ or µg g$^{-1}$) ± s.e.* | | | | | |
|---|---|---|---|---|---|---|
| | *Tumour bearing Time (min)* | | | *Non-tumour bearing Time (min)* | | |
| | *5* | *15* | *30* | *5* | *15* | *30* |
| Plasma | 109.0 ± 4.6 | 50.1 ± 5.3 | 32.8 ± 8.7 | 133.0 ± 18.9 | 49.0 ± 11.7 | 40.5 ± 3.5 |
| Liver | 24.5 ± 1.4 | 22.9 ± 2.9 | 21.7 ± 5.9 | 25.4 ± 2.5 | 26.9 ± 6.9 | 25.8 ± 4.1 |
| Lung | 28.3 ± 2.3 | 17.6 ± 3.2 | 14.4 ± 3.7 | 36.4 ± 4.8 | 18.2 ± 4.7 | 14.4 ± 1.5 |
| Tumour | 11.0 ± 1.7 | 10.3 ± 1.8 | 11.5 ± 2.4 | | | |

Both groups of mice received A5B7–F(ab')$_2$:CPG2 (1,000 U kg$^{-1}$, i.v.) 6 days before the prodrug. A delay of 6 days was required for antibody–enzyme conjugate to clear from plasma. If less than 6 days was allowed before prodrug administration, mice died from non-localised active drug production. Four mice were used per time point. The samples were not pooled but were analysed separately.

drug in the presence of the conjugate (plasma peak level = 6.8 $\mu g\ ml^{-1}$; Bagshawe *et al.*, 1988). The plasma protein binding was high for both prodrug (92%) and active drug (96%).

The antibody–enzyme conjugates W14–$F(ab')_2$ : CPG2 and A5B7–$F(ab')_2$ :CPG2 were shown to localise preferentially at the tumour site and to retain enzyme activity, for at least 6 days. The distribution of these conjugates has been studied previously by radiolabelling the enzyme and conjugating it to the fragmented antibody (Melton *et al.*, 1990; Sharma *et al.*, 1990). The advantage of the novel method described here is that a measure of the activity of the localised enzyme is obtained.

When prodrug was given to nude mice bearing human CC3 choriocarcinoma xenografts which had received antibody–enzyme conjugate 72 h earlier, active drug was detectable in plasma and tissues within 5 min. Although it was shown that the tumour had the highest level of enzyme activity, it did not have the highest level of active drug. However, it was the only tissue that activated all the available prodrug. A study of the prodrug distribution (Figure 7a) demonstrated that tumour had the lowest level of prodrug. The amount of active drug generated by tumour was therefore limited by the amount of prodrug and not the enzyme. From the results it appears that enzyme kinetics may play an important role in relation to prodrug concentration at the tumour site. The enzyme in plasma and tissues did not activate all the prodrug received at these sites. This was probably due to the relatively high levels of prodrug and low levels of enzyme, since tumour located enzyme activated all the available prodrug. This would indicate that it may be possible to use larger doses of prodrug in order to achieve greater production of active drug at tumour sites without comparable enhancement of active drug production elsewhere.

Further studies were undertaken in order to ascertain whether the active drug found in non-tumour tissues was produced at the tumour site and then released into plasma or was due to the non-specific presence of the antibody–enzyme conjugate in plasma and tissues, which led to activation of prodrug at these sites. Mice bearing human LS174T colon adenocarcinoma xenografts were compared with non-tumour bearing nude mice. Both sets of mice received A5B7–$F(ab')_2$: CPG2, followed by prodrug 6 days later. The results, when compared to the active drug levels expected in the absence of conjugate, indicated that the presence of antibody–enzyme conjugate at non-tumour sites was responsible for activation of prodrug in plasma and other tissues. The enzyme has a very high affinity for prodrug (Bagshawe *et al.*, 1988) and although the enzyme levels in tissues other than tumour and lung were comparatively low, they were still capable of activating prodrug.

It was shown previously that the prodrug 4-(*bis* (2-chloroethyl) amino) benzoyl-L-glutamic acid had very little cytotoxicity against the human JAR choriocarcinoma and LS174T colonic cell lines in culture (Springer *et al.*, 1990). On the other hand the activated prodrug was over 100 times more cytotoxic to JAR cells. It has also been shown previously that although CC3 tumours have proved resistant to a wide range of conventional cytotoxic agents, a marked inhibition of growth of CC3 tumours in nude mice occurred with a single course of treatment with the two phase prodrug therapy described here (Bagshawe *et al.*, 1988). In the present study we have demonstrated that the prodrug is converted by antibody–enzyme conjugate *in vivo* immediately to its active derivative, benzoic acid mustard. The low concentration of residual antibody–enzyme conjugate which remains in plasma and tissues is able to activate the prodrug. This observation confirms the need to confine enzyme activity to tumour sites as rigorously as possible. A method of inactivating enzyme in plasma without inactivating enzyme at tumour sites has been developed in anticipation of these findings (Bagshawe, 1989; Sharma *et al.*, 1990).

We thank the Cancer Research Campaign for grant support. We are grateful to Miss J. Boden and Mr R. Boden for skilled technical assistance. We also wish to thank Dr John Baer for discussions on HPLC analysis.

### References

BAGSHAWE, K.D. (1987). Antibody directed enzymes revive anti-cancer prodrugs concept. *Br. J. Cancer*, **56**, 531.

BAGSHAWE, K.D. (1989). Towards generating cytotoxic agents at cancer sites. *Br. J. Cancer*, **60**, 275.

BAGSHAWE, K.D., SPRINGER, C.J., SEARLE, F. & 4 others (1988). A cytotoxic agent can be generated selectively at cancer sites. *Br. J. Cancer*, **58**, 700.

HARWOOD, P.J., BRITTON, D.W., SOUTHALL, P.J., BOXER, G.M., RAWLINS, G. & ROGERS, G.T. (1986). Mapping epitope characteristics on carcinoembryonic antigen. *Br. J. Cancer*, **54**, 75.

JOHNSON, V.G., SCHLOM, J., PATERSON, A.J., BENNETT, J., MAGNANI, J.L. & COLCHER, D. (1986). Analysis of a human tumour-associated glycoprotein (TAG-72) identified by monoclonal antibody B72.3. *Cancer Res.*, **46**, 850.

JOHNSTONE, A. & WOOLARD, R.C. (1983). STRIPE: an interactive computer program for the analysis of drug pharmacokinetics. *J. Pharmacol. Methods*, **9**, 193.

MELTON, R.G., SEARLE, F., SHERWOOD, R.F., BAGSHAWE, K.D. & BODEN, J.A. (1990). The potential of carboxypeptidase G2 antibody conjugate as antitumour agents. II. *In vivo* localising and clearance properties in a choriocarcinoma model. *Br. J. Cancer*, **61**, 420.

PEDLEY, R.B., BODEN, J., KEEP, P.A., HARWOOD, P.J., GREEN, A.J. & ROGERS, G.T. (1987). Relationship between tumour size and uptake of radiolabelled anti-CEA in a colon tumour xenograft. *Eur. J. Nucl. Med.*, **13**, 197.

ROSS, W.C.J. & WARWICK, G.P. (1955). Reduction of cytotoxic compounds by hydrazine and by the xanthine oxidase system. *Nature*, **176**, 298.

SEARLE, F., BIER, C., BUCKLEY, R.G. & 6 others (1986). The potential of carboxypeptidase G2 antibody conjugates as anti-tumour agents. I. Preparation of antihuman chorionic gonadotrophin carboxypeptidase G2 and cytotoxicity of the conjugates against JAR choriocarcinoma cells *in vitro*. *Br. J. Cancer*, **53**, 377.

SEARLE, F., BODEN, J.A., LEWIS, J.C.M. & BAGSHAWE, K.D. (1981). A human choricarcinoma xenograft in nude mice: A model for the study of the antibody localization. *Br. J. Cancer*, **44**, 137.

SHARMA, S.K., BAGSHAWE, K.D., BURKE, P.J., BODEN, R.W. & ROGERS, G.T. (1990). Inactivation and clearance of an anti-CEA carboxypeptidase G2 conjugate in blood after localisation in a xenograft model. *Br. J. Cancer*, **61**, 659.

SHERWOOD, R.F., MELTON, R.G., ALWAN, S.M. & HUGHES, P. (1985). Purification and properties of carboxypeptidase G2 from *Pseudomonas* sp strain RS-16. Use of a novel triazine dye affinity method. *Eur. J. Biochem.*, **148**, 447.

SMITH, J.F.M. & THIJSSEN, H.H.W. (1986). Spatial control of drug action: theoretical considerations on the pharmacokinetics of the target-aimed drug. In *Rate-controlled Drug Administration and Action*, Struyker-Boudier, H.A.J. (ed.) p. 83. CRC Press: Boca Raton, FL.

SPRINGER, C.J., ANTONIW, P., BAGSHAWE, K.D., SEARLE, F., BISSET, G.M.F. & JARMAN, M. (1990). Novel prodrugs which are activated to cytotoxic alkylating agents by carboxypeptidase G2. *J. Med. Chem.*, **33**, 677.